# CDK1: beyond cell cycle regulation

**DOI:** 10.18632/aging.101348

**Published:** 2017-12-14

**Authors:** Hongwei Liao, Fang Ji, Songmin Ying

**Affiliations:** Department of Respiratory and Critical Care Medicine of Second Affiliated Hospital, Zhejiang University School of Medicine, Hangzhou 310058, China

**Keywords:** DNA replication, cell cycle, CDK1, stalled fork, DNA fibre

Cyclin-dependent kinase 1 (CDK1) has recently been shown to promote replicative DNA synthesis and may contribute to chemoresistance. These findings provide novel insights into coordination between cell cycle regulation and DNA replication in the maintenance of genomic stability.

Precise DNA replication is essential in the maintenance of genomic stability. During each round of cell cycle, more than billions of base pairs must be replicated timely and faithfully, depending on the integrity of every individual replication fork. Replication forks are often challenged by impediments including transcription complexes, DNA lesions, and secondary DNA structures, leading to fork slowdown/stalling [1]. In cancer cells, replication forks are additionally challenged by oncogene-induced replication stress, as well as by chemo- or radio-therapeutic perturbations-induced stress. To ensure genome duplication is completed before the initiation of mitosis, a plethora of mechanisms are employed by cells to deal with replicative challenges, maintaining the function and integrity of replication forks. In normal cells and tissues, these replication maintenance mechanisms safeguard the genome and represent the natural barriers to diseases correlated with genome instability, including cancer. Paradoxically, the fork maintenance mechanisms can also be exploited by cancer cells to acquire resistance to chemotherapeutic agents that are based on interfering with DNA replication and causing fork stalling [2]. Taken together, elucidation of how cells protect their delicate replication forks not only will expand our knowledge of carcinogenesis, but also holds plausible promise for improving current chemotherapies.

During the last decade, a great progress has been made in the molecular dissection of fork maintenance mechanisms. Apart from those replicasome components that are dogmatically accepted, a number of new factors are identified to be required for efficient replication progression in the absence or presence of replication perturbations. Among these factors, some are constitutive factors that are essential for the function of replication forks, such as downstream neighbor of SON (DONSON) [3]. Some are protective factors that are specifically needed under pathological conditions, in which they operate to prevent fork collapse by detecting and limiting replication stress. These fork-protective factors are represented by Fanconi anemia proteins and homologous recombination (HR) proteins, which are capable to stabilize RAD51 nucleoprotein filaments at the stalled replication forks. These functions suppress nascent DNA strand degradation and promote continuous DNA synthesis. Moreover, some checkpoint-activating proteins are also discovered to be essential for robust genome replication [4]. In light of those extensive studies on replication fork maintenance, we now have collective knowledge of many factors that operate to maintain genome stability. However, because nearly all the studies are only looking into individual factors of interest, the interconnections among these factors and how they are coordinated are constantly out of the spotlight. In our recently published work, we seem to take a preliminary glimpse at a potential DNA replication coordinator that has long been considered essential in cell cycle regulation, cyclin-dependent kinase 1 (CDK1) [5].

As a protein kinase, CDK1 is more than a cell cycle regulator as it was originally identified. It has been shown to carry versatile functions during the last decade. From cell cycle control to DNA damage repair, CDK1 intimately involves in many cellular events that are vital for cell survival. The activity of CDK1 oscillates during each cell cycle, which peaks at the G2/M phase and remains low at G1/S phase. Interestingly, the remaining expression of CDK1 in S phase is still comparable to CDK2, suggesting a potential role for CDK1 in DNA replication [6]. Roles for CDK1 in replication remain poorly understood mainly because of two reasons. First, genetic interference with CDK1 could cause G2/M arrest, rendering it difficult to study S phase events; Second reason was due to the lack of a specific CDK1 inhibitor with minimal off-target effect.

By harnessing the new-generation CDK1 inhibitors with high specificity, we were allowed to manipulate CDK1 activity in a very short time period without affecting the cell cycle progression and causing promiscuous off-target effects. We inhibited CDK1 shortly and demonstrated that CDK1 was required for efficient DNA replication and prevention of replication-associated DNA damage. Since CDK1 is a protein kinase that can phosphorylate numerous targets, it is probably that CDK1 promotes replicative DNA synthesis via functional phosphorylation of multiple downstream effectors. Future studies need to combine CDK1 inhibitors and phosphoproteomics to profile CDK1-dependent phosphorylation changes of all the potential effectors, which may lead to better understanding of how CDK1 is involved in coordination of DNA replication. Given that CDK1 also participates in checkpoint activation and HR-dependent DNA repair, we hypothesized that inhibition of CDK1 may enhance the killing effects of replication-toxic agents by permitting accumulation of excessive DNA damage. In agreement, we showed that comprised CDK1 activity sensitized cancer cells to multiple chemotherapeutic drugs that act through disrupting DNA replication.

**Figure 1:**
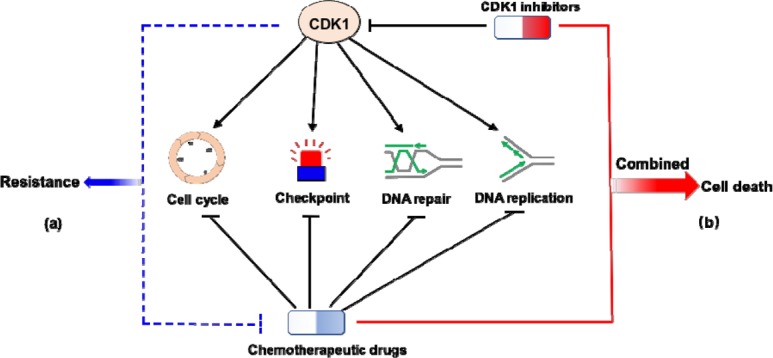
Functional versitility and pharmacological relevance of CDK1 CDK1 promotes multiple biological processes that are critical for cell survival, including G2/M transition, checkpoint activation, DNA repair, and DNA replication as we propose. Its activities in these processes compromise the efficacy of chemotherapeutic drugs and may contribute to chemoresistance (**a**). Hence, combining pharmological inhibition of CDK1 with conventionl anti-cancer drugs will potentially enhance toxicity to cancer cells and lead to cell death (**b**).

In summary, our study has uncovered an unanticipated role for CDK1 in DNA replication, adding to our already very complex understanding of the replication fork maintenance mechanisms. In addition, based on our data and other functions of CDK1 reported before, pharmacological inhibition of CDK1 represents a promising strategy in combined cancer treatment (Figure 1). Therefore from a clinical point of view, it is imperative to further unveil the multifaceted role of CDK1 in various biological processes.
